# Peer education programme to improve adolescent sexual and reproductive health in Rwanda

**DOI:** 10.4102/jphia.v16i1.1342

**Published:** 2025-10-08

**Authors:** Aimable Nkurunziza, Michael Habtu, Germaine Tuyisenge, Nadja Van Endert, Godfrey Katende, Assumpta Yamuragiye, Justine Bagirisano, Jean B.H. Hitayezu, Olive Tengera, Edward Rwagasore

**Affiliations:** 1 School of Nursing, College of Medicine and Health Sciences, University of Rwanda, Kigali, Rwanda; 2 Arthur Labatt Family School of Nursing, Faculty of Health Sciences, Western University, London, ON, Canada; 3 School of Nursing, Faculty of Education and Professional Studies, Nipissing University, North Bay, Canada; 4 School of Public Health, College of Medicine and Health Sciences, University of Rwanda, Kigali, Rwanda; 5 Faculty of Epidemiology and Public Health, London School of Hygiene and Tropical Medicine, London, United Kingdom; 6 Faculty of Health Sciences, Simon Fraser University, Vancouver, BC, Canada; 7 Department of Midwifery, University Colleges Leuven-Limburg (UCLL), Genk, Belguim; 8 School of Nursing, Aga Khan University, Kampala, Uganda; 9 School of Health Sciences, College of Medicine and Health Sciences, University of Rwanda, Kigali, Rwanda; 10 Department of Midwifery, College of Medicine and Health Sciences, University of Rwanda, Kigali, Rwanda; 11 Health Development Initiative, Kigali, Rwanda

**Keywords:** high school, adolescents, peer education programme, sexual and reproductive health, Rwanda

## Abstract

**Background:**

A peer education programme was developed in response to the tendency of high school students in Rwanda to seek sexual and reproductive health information from peers who are often inadequately informed.

**Aim:**

To assess the effect of Sexual and Reproductive Health Peer Education Programme (SRH PEP) on knowledge and the attitudes of SRH among high school adolescents in Rwanda.

**Setting:**

The study was conducted at selected high schools in Rwanda.

**Methods:**

This pre-test and post-test design study was conducted in two selected high schools. The pre-test data were collected in February 2020, followed by the post-test data in May 2022. A total of 536 students participated in this study. The effect on SRH knowledge and attitudes was measured using a paired *t*-test.

**Results:**

Of the total 536 questionnaires administered, only 508 were well completed (response rate of 94.7%). After the intervention, there was an increase in knowledge and attitude regarding SRH (*M* = 15.87 vs 19.9, *p* < 0.001; *M* = 7.95 vs 10.66, *p* < 0.001, respectively). The proportion of correct responses to knowledge and attitude was also significantly improved after the intervention (*p* < 0.05).

**Conclusion:**

This study underscored the pivotal role of peer-led SRH education programmes as an effective modality for educating adolescents and young adults about SRH. There is a need for integrating structured peer education initiatives into school-based programmes to ensure adolescents receive accurate and reliable SRH information.

**Contribution:**

This study contributes to the field of adolescent SRH by demonstrating the effectiveness of peer education programmes (PEPs) in enhancing knowledge and attitudes among high school students.

## Introduction

### Background

Sexual and reproductive health and rights (SRHR) is an essential and integral component of universal health coverage that countries around the globe need to consider for attainment of the sustainable development goals (SDGs). Limited access to sexual and reproductive health (SRH) among adolescents has been reported as a challenge in many countries worldwide.^1^ Lack of or inadequate SRH knowledge among adolescents has severe consequences, including but not limited to increased teenage pregnancies, early marriage and poor decision-making.^2^

Worldwide, teenage pregnancy remains a major concern with variations in different countries, including sub-Saharan Africa (SSA) as the most affected.^3,4,5,6,7,8^ The East African region accounts for 25% of the SSAs prevalence.^9^ In Rwanda, one of the East African countries, pregnancies among young girls between 10 years and 18 years continue to be reported, with the eastern province having the highest prevalence.^10^ Teenage pregnancy consequently leads to school dropouts, extreme poverty, complicated pregnancies and associated risks, malnutrition as well as mental health consequences.^11^

A recent study conducted in Rwanda about adolescent reproductive health and sources of information revealed that sexuality as a topic is considered taboo in Rwandan culture.^12^ However, another study that was conducted to explore enabling and preventive factors to access to the SRH among adolescents revealed that 5% of female and 11% of male adolescents became engaged in sexual activity before the age of 15 years.^13^ This high prevalence of teenage pregnancies and adolescents’ behaviour of engaging in sexual activities suggests a need to address the problem but also calls for implementing different interventions to provide and increase access to quality sex education.

Since 2016, Rwanda has been implementing a comprehensive SRH education in primary and secondary schools together with other different interventions such as teaching in and out schooling and mobile for reproductive health (m4RH) that delivered reliable SRH information to early adolescents.^11^ However, from statistics, it appears that most of these interventions seem to be less effective with little or no significant outcomes.^14^ This might be argued that many of the adolescents seek information from their peers, who might also be equipped with little or incorrect knowledge about SRH.^15^ This, therefore, led to the development and implementation of a Sexual and Reproductive Health Peer Education Programme (SRH PEP) conducted in the selected district of Rwanda. The selection of the district was based on the current country’s highest prevalence of teenage pregnancies in Eastern Province.^16^ The SRH PEP was implemented to equip adolescents with knowledge and the right attitudes about SRH in schools but also to contribute to addressing the problem. The outcomes of implementing the SRH PEP were also evaluated.

## Research methods and design

### Study design

This study utilised both pre-intervention and post-intervention designs. A baseline (pre-test) survey was conducted on SRH knowledge and attitudes among adolescents before the implementation of the intervention. A post-test survey was conducted after 26 months of the implementation of the intervention.

### Description of the intervention

The SRH PEP Manual was developed in 2019 in collaboration with the University of Rwanda (UR) and University College Leuven-Limberg (UCLL) staff.^16^ The development of the Manual undertook several steps to the final implementable Manual. The literature review process was conducted to understand the available evidence around the implementation of the SRH PEP. A local needs assessment was then conducted to ensure that the intervention was tailored to the local and adolescents’ needs. A networking event was also organised with different stakeholders, including schools’ leadership, teachers, community leaders, religious people and healthcare providers among others. The purpose of this event was to gain a comprehensive understanding of existing SRH programmes in the district or schools, the challenges faced during their implementation in the schools and the communities. The networking event was also used as an opportunity to connect with the stakeholders for programme sustainability. Baseline surveys were then conducted using mixed methods. The quantitative approach was used to examine adolescents’ knowledge and their attitudes regarding SRH, while the qualitative approach was conducted through six focus group discussions (FGDs) to obtain a deeper understanding of adolescents’ SRH needs. These were analysed and used to inform the development of the SRH PEP Manual. Lastly, the team from UR visited UCLL to discuss SRH issues and outreach programmes in Rwanda.

During the implementation of the SRH PEP, the empowerment model developed by the research team guided the process.^16^ With the use of the SRH PEP Manual, a 6-day workshop was conducted to train selected students to become peer educators (PEs). The goal of the workshop was to equip PEs with enough SRH knowledge and improve their personal development and group communication skills. During the workshop, evidence-based experimental learning and interactive methodologies were used. These strategies were also used to help PEs discover new information about themselves. To ensure that PEs were actively involved and retained information, active learning strategies such as role plays, simulations and educational games were utilised.^17^

We conducted two different trainings (training module A for UR midwifery students as facilitators and train-the-trainer module B for PEs).^18^ Six of the trained facilitators (midwifery students) and three staff members of UR midwifery trained the selected students at three secondary schools. In each school, we selected 12 students as PEs (six males and six females). In addition, we trained their teachers who, at the time, showed interest in learning more about SRH issues and also in implementing the SRH Module. This was not previously planned but, in the end, added value to the project for sustainability. These teachers then took up the mentorship role of the students during the project implementation. After we completed the training, we then embarked on creating a WhatsApp group among the project team, trained teachers and schools’ authorities to ensure constant communication. The project team contacted the trained peers by phone through their teachers and authorities at least twice a month to monitor any challenges and propose immediate solutions. Focus group discussions were conducted with PEs to explore their experiences as evidence that PEs improved the programme.^18^ After 26 months of implementing the SRH PEP, we again assessed their knowledge and attitudes.

### Study participants

The SRH PEP was implemented in three secondary-level high schools. However, due to unforeseen challenges faced by one school in the implementation of the project – with mentors’ relocation to other schools and repatriation of PEs to Burundi from the refugee camp,^19^ the post-intervention was only conducted in two schools. Therefore, 536 students aged between 13 years and 24 years from secondary school levels 2, 3, 5 and 6 participated in both the pre-survey and the post-survey. Students from secondary levels 1 and 4 were excluded because they were new to the schools and programme from primary level and other schools, respectively. The findings from the pre-test evaluations from the third school were not considered in this manuscript. After excluding the incomplete data from the total 536 students who had participated in the baseline, 508 students enrolled in the programme were included in the analysis.

### Data collection instrument and procedures

A self-administered questionnaire, the World Health Organisation’s (WHO) ‘Illustrated Questionnaire for Interview Surveys with Young People’ was adapted and utilised as a data collection tool for this study.^20^ The questionnaire consisted of 23 questions/statements on knowledge with different subscales such as four items for sexual health, eleven items on contraceptive methods and eight items on human immunodeficiency virus (HIV) and/or acquired immunodeficiency syndrome (AIDS) and sexually transmitted diseases (STDs). A score of one (1) was allocated to the correct response, whereas a score of zero (0) was given to the incorrect response. Similarly, there were 13 items for the attitudes, and a Score 1 was assigned for the positive attitude, whereas a Score 0 for the negative attitude. Higher total scores implied better knowledge and attitudes. Six students from each school were recruited for the pilot of the instrument. The findings from the pilot study yielded Cronbach’s alpha of 0.85. The students also provided their feedback to make the tool easier to complete. During the pilot study, the students were not included in the study. The final corrected tool was developed from the feedback obtained from the assessed students. Before the implementation of the project, the questionnaire was administered to all (*N* = 536) students.

### Data analysis

Data were analysed using the Statistical Package for the Social Sciences (SPSS) version 25 (SPSS Inc., Chicago, IL, United States). Descriptive statistics were conducted, with frequencies and percentages used for categorical variables, and means with standard deviations calculated for continuous variables. McNemar test was used to compare categorical knowledge and attitude variables between pre-test and post-test intervention. The score changes of knowledge and attitudes as continuous variables between pre-test and post-test interventions were evaluated using paired *t*-test. A *p*-value of less than 0.05 was considered as statistically significant.

### Ethical considerations

Ethical clearance to conduct this study was obtained from the University of Rwanda College of Medicine and Health Sciences Institutional Review Board (No. 158/CMHS IRB/2019). Participants aged less than 18 years old signed assent forms, and their legal guardians signed the informed consents, while those aged 18 years and more signed the informed consents.

## Results

### Demographic attributes of respondents

Slightly more than half (53.7%) of the respondents were in the age range of 16 years to 18 years, with significantly more females in this age group (*p* = 0.001). There was nearly an equal distribution of participants for all the classes involved. Large proportion (49.0%) of students were from Protestants’ religious affiliation. About 9% were taking alcohol with more among males (*p* < 0.001), while smoking was only 0.4%. Approximately half of the participants (48.9%) had never engaged in conversations with their fathers about sexual matters, compared with 30.5% who had not discussed such topics with their mothers. Among the respondents, 13.8% reported having had sexual intercourse and, of these, the majority (70.6%) indicated that they had used contraceptives (Table 1).

**TABLE 1:** Selected attributes of respondents (*N* = 508).

Attributes	Total		Males (*n* = 259)		Females (*n* = 249)		*p*-value*
	*n*	%	*n*	%	*n*	%	
**Age (years)**	-	-	-	-	-	-	0.001
13–15	50	9.8	22	8.5	28	11.2	-
16–18	273	53.7	126	48.6	147	59.0	-
19–21	175	34.4	101	39.0	74	29.7	-
22–24	10	2.0	10	3.9	0	0.0	-
**Study year**	-	-	-	-	-	-	0.262
S2	142	28.0	65	25.1	77	30.9	-
S3	127	25.0	69	26.6	58	23.3	-
S5	128	25.2	72	27.8	56	22.5	-
S6	111	21.9	53	20.5	58	23.3	-
**Religion**	-	-	-	-	-	-	0.001
None	11	2.2	11	4.2	0	0.0	-
Catholic	166	32.7	90	34.7	76	30.5	-
Protestant	249	49.0	112	43.2	137	55.0	-
Muslim	22	4.3	17	6.6	5	2.0	-
Adventist	42	8.3	20	7.7	22	8.8	-
Others	18	3.5	9	3.5	9	3.6	-
**Alcohol**	-	-	-	-	-	-	< 0.001
Yes	46	9.1	44	17.0	2	0.8	-
No	462	90.9	215	83.0	247	99.2	-
**Smoking**	-	-	-	-	-	-	0.165
Yes	2	0.4	2	0.8	0	0.0	-
No	506	99.6	257	99.2	249	100.0	-
**Discuss sex-related matters with father (*n* = 497)**	-	-	-	-	-	-	0.098
Often	75	15.1	36	14.3	39	15.9	-
Occasionally	179	36.0	81	32.1	98	40.0	-
Never	243	48.9	135	53.6	108	44.1	-
**Discuss sex-related matters with mother (*n* = 501)**	-	-	-	-	-	-	< 0.001
Often	197	39.3	48	18.8	149	60.6	-
Occasionally	151	30.1	83	32.5	68	27.6	-
Never	153	30.5	124	48.6	29	11.8	-
**Ever experienced sexual intercourse**	-	-	-	-	-	-	0.001
Yes	70	13.8	49	18.9	21	8.4	-
No	438	86.2	210	81.1	228	91.6	-
**Use of contraceptive (*n* = 70)**	-	-	-	-	-	-	0.606
Yes	48	70.6	33	68.8	15	75.0	-
No	20	29.4	15	31.3	5	25.0	-

* , Chi-square test was used to compare the proportion between males and females.

### Knowledge on sexual health, contraceptives and HIV and/or AIDS and STDs according to pre- and post-Sexual and Reproductive Health Peer Education Programme

Table 2 demonstrates the responses of adolescents to knowledge questions provided pre-education and post-education interventions. The knowledge was divided into three subscales: sexual health, contraceptive methods and HIV and/or AIDS and STDs. The knowledge had significantly improved (*p* < 0.01) for all statements except for two questions under the subscale of HIV and/or AIDS and STDs, which were cure of HIV and/or AIDS and availability of simple test for HIV (Table 2).

**TABLE 2:** Knowledge on sexual health, contraceptives and HIV and/or AIDS and STDs according to pre- and post-Sexual and Reproductive Health Peer Education Programmes.

Questions or statements	Correct responses				*p*-value*
	Pre-intervention		Post-intervention		
	*n*	%	*n*	%	
**Knowledge on sexual health**					
A girl or woman can get pregnant on the very first time that she has sexual intercourse.	359	70.7	414	81.5	< 0.001
A girl or woman stops growing after she has had sexual intercourse for the first time.	332	65.4	418	82.3	< 0.001
Masturbation causes serious damage to health.	452	89.0	495	97.4	< 0.001
A girl or woman is most likely to get pregnant if she has sexual intercourse half way between her periods.	154	30.3	274	53.9	< 0.001
**Knowledge on contraceptive methods**					
Women can take a pill every day.	371	73.0	473	93.1	< 0.001
Women can have an injection every 2 weeks or every 3 months.	410	80.7	500	98.4	< 0.001
A man can put a rubber device on his penis before intercourse.	377	74.2	505	99.4	< 0.001
A woman can take pills soon after intercourse.	403	79.3	504	99.2	< 0.001
A man can pull out of a woman before climax.	406	79.9	483	95.1	< 0.001
A couple can avoid sex on days when pregnancy is most likely to occur.	452	89.0	500	98.4	< 0.001
Whether knowing IUD as a contraceptive device.	256	50.4	374	73.6	< 0.001
Whether knowing implant as contraceptive method.	310	61.0	395	77.8	< 0.001
Whether knowing jelly or foam products.	88	17.3	262	51.6	< 0.001
Whether knowing female sterilisation as contraceptive.	276	54.3	364	71.7	< 0.001
Whether knowing male sterilisation as contraceptive.	215	42.3	351	69.1	< 0.001
**Knowledge towards HIV and/or AIDS and STDs**					
Have you heard of HIV and/or AIDS diseases?	495	97.4	508	100.0	NA
It is possible to cure AIDS.	309	60.8	333	65.6	0.128
A person with HIV always looks emaciated or unhealthy in some way.	380	74.8	417	82.1	0.005
People can take a simple test to find out whether they have HIV.	479	94.3	488	96.1	0.243
Apart from HIV and/or AIDS, there are other diseases that men and women can catch by having sexual intercourse without any protection.	488	96.1	508	100.0	NA
Vaginal discharge is sign or symptom when a man or woman is infected.	251	49.4	508	100.0	NA
Pain during urination is sign or symptom when a man or woman is infected.	440	86.6	473	93.1	0.001
Ulcers or sores are the signs or symptoms when a man or woman is infected.	359	70.7	406	79.9	0.001

NA, not applicable as cell number must be greater than 1; IUD, intrauterine device; HIV, human immunodeficiency virus; AIDS, acquired immune deficiency syndrome; STDs, sexually transmitted diseases

* , McNemar Bowker test was used.

### Attitudes towards contraceptive methods according to pre- and post-Sexual and Reproductive Health Peer Education Programmes

Table 3 provides the responses of adolescents to the attitudes on contraceptive methods during the pre-education and post-education interventions. The respondents demonstrated significant increase in the positive attitudes to all the statements after implementing the SRH PEP (*p* < 0.001).

**TABLE 3:** The attitudes towards contraceptive methods according to pre- and post-Sexual and Reproductive Health Peer Education Programme.

Questions or statements	Positive attitude				*p*-value*
	Pre-intervention		Post-intervention		
	*n*	%	*n*	%	
Condoms are effective method of preventing pregnancy.	301	59.3	365	71.9	< 0.001
Condoms can be used more than once.	394	77.6	463	91.1	< 0.001
A girl can suggest to her boyfriend that he use a condom.	373	73.4	444	87.4	< 0.001
A boy can suggest to his girlfriend that he use a condom.	438	86.2	465	91.5	0.013
Condoms are an effective way of protecting against HIV and/or AIDS.	396	78.0	466	91.7	< 0.001
Condoms are suitable for casual relationships.	252	49.6	372	73.2	< 0.001
Condoms are suitable for steady, loving relationships.	255	50.2	410	80.7	< 0.001
It would be too embarrassing for someone like me to purchase or obtain condoms.	282	55.5	449	88.4	< 0.001
If a girl suggested using condoms to her partner, it would mean that she did not trust him.	370	72.8	485	95.5	< 0.001
Condoms reduce sexual pleasure.	129	25.4	259	51.0	< 0.001
Condoms can slip off the man and disappear inside the woman’s body.	46	9.1	301	59.3	< 0.001
If unmarried couples want to have sexual intercourse before marriage, they should use condoms.	426	83.9	467	91.9	< 0.001
Condoms are an effective way of protecting against sexually transmitted diseases.	377	74.2	468	92.1	< 0.001

HIV, human immunodeficiency virus; AIDS, acquired immune deficiency syndrome.

* , McNemar Bowker test was used.

### Effect of Sexual and Reproductive Health Peer Education Programme on knowledge and the attitudes towards sexual health, contraceptives and HIV and/or AIDS and STDs

Table 4 indicates that the mean overall knowledge scores among adolescent students had significantly increased from 15.87 before the intervention to 19.59 after the intervention (*p* < 0.001). Similarly, the mean knowledge score for each subscale had improved significantly after the SRH PEP (*p* < 0.001). A similar result was also observed for the mean attitude scores, where it significantly improved from 7.95 in the pre-intervention to 10.66 in the post-intervention (*p* < 0.001).

**TABLE 4:** Effect of Sexual and Reproductive Health Peer Education Programme on knowledge and the attitudes towards sexual health, contraceptives and HIV and/or AIDS and STDs.

Knowledge and attitude scores	Pre-test		Post-test		Paired *t*-test	*p*-value
	Mean	s.d.	Mean	s.d.		
Knowledge score on sexual health	2.55	1.00	3.15	0.81	−10.58	< 0.001
Knowledge score on contraceptive methods	7.02	2.17	9.27	1.87	−17.87	< 0.001
Knowledge score on HIV and/or AIDS and STDs	6.30	1.16	7.17	0.84	−13.70	< 0.001
Overall knowledge score on sexual health, contraceptive methods and HIV and/or AIDS and STDs	15.87	3.11	19.59	2.43	−21.72	< 0.001
Attitudes score on contraceptive methods	7.95	1.86	10.66	1.50	−25.62	< 0.001

STDs, sexually transmitted diseases; IUD, intrauterine device; HIV, human immunodeficiency virus; AIDS, acquired immune deficiency syndrome; s.d., standard deviation; AIDS, acquired immune deficiency syndrome.

### Knowledge and the attitude scores based on socio-demographic characteristics

The attitude score on contraceptive was significantly higher among male students than female students (*p* = 0.005) before intervention; however, this difference was not significant after the intervention (*p* = 0.986). Students from Seniors 5 and 6 had significantly (*p* < 0.001) higher knowledge scores compared to those in Seniors 3 and 4 in both the pre-intervention and the post-intervention. After the intervention, the overall knowledge score was significantly higher among adolescents aged from 22 years to 24 years (*p* = 0.022), but there were no significant differences before the intervention (Table 5).

**TABLE 5:** Knowledge and the attitude scores based on socio-demographic characteristics.

Characteristics	Pre-test				Post-test			
	Overall knowledge score		Attitude score		Overall knowledge score		Attitude score	
	Mean	*p*-value	Mean	*p*-value	Mean	*p*-value	Mean	*p*-value
**Age (years)**	-	0.156	-	0.559	-	0.022	-	0.461
13–15	15.20	-	8.26	-	19.04	-	10.84	-
16–18	15.79	-	7.86	-	19.42	-	10.68	-
19–21	16.22	-	7.99	-	19.97	-	10.55	-
22–24	15.20	-	8.00	-	20.50	-	11.10	-
**Sex**	-	0.940	-	0.005	-	0.840	-	0.986
Male	15.88	-	8.18	-	19.61	-	10.66	-
Female	15.86	-	7.71	-	19.57	-	10.66	-
**Study year**	-	0.001	-	0.903	-	< 0.001	-	0.412
S3	15.57	-	7.96	-	18.18	-	10.80	-
S4	15.14	-	7.87	-	20.17	-	10.72	-
S5	16.59	-	8.04	-	19.52	-	10.52	-
S6	16.26	-	7.93	-	20.83	-	10.58	-
**Religion**	-	0.929	-	0.480	-	0.766	-	0.262
None	15.55	-	7.27	-	20.36	-	10.09	-
Catholic	15.86	-	7.97	-	19.76	-	10.64	-
Protestant	15.89	-	7.89	-	19.49	-	10.74	-
Muslim	16.50	-	8.59	-	19.50	-	10.32	-
Adventist	15.60	-	8.07	-	19.43	-	10.36	-
Others	15.83	-	7.94	-	19.44	-	11.06	-
**Alcohol**	-	0.123	-	0.309	-	0.812	-	0.291
Yes	16.54	-	8.22	-	19.67	-	10.43	-
No	15.80	-	7.92	-	19.58	-	10.68	-
**Smoking**	-	0.154	-	0.119	-	0.131	-	0.882
Yes	19.00	-	10.00	-	17.00	-	10.50	-
No	15.86	-	7.94	-	19.60	-	10.66	-
**Discuss sex-related matters with father (*n* = 497)**	-	0.644	-	0.705	-	0.644	-	0.389
Often	15.93	-	7.91	-	19.84	-	10.68	-
Occasionally	16.02	-	7.89	-	19.56	-	10.53	-
Never	15.74	-	8.03	-	19.55	-	10.73	-
**Discuss sex-related matters with mother (*n* = 501)**	-	0.168	-	0.707	-	0.943	-	0.450
Often	16.19	-	7.94	-	19.64	-	10.56	-
Occasionally	15.70	-	8.05	-	19.55	-	10.77	-
Never	15.62	-	7.87	-	19.61	-	10.67	-

Note: For comparing the means, independent *t*-test and analysis of variance (ANOVA) were used.

## Discussion

The purpose of this study was to evaluate the effect of the SRH PEP on knowledge and the attitudes of SRH among high school adolescents. The findings from the current study revealed that the knowledge on sexual health, contraceptive methods and HIV and/or AIDS and STDs had significantly improved after implementing SRH PEP. Young students’ mean overall knowledge score of SRH significantly rose from 15.87 prior to intervention to 19.59 following the SRH PEP. Similar findings were seen for the mean attitude score, which revealed a considerable increase from 7.95 prior to intervention to 10.66 following intervention.

Despite the prolific implementation of peer education programmes (PEPs) globally, reviews found that there is a mixture of the PEP outcomes – some lead to positive outcomes and others do not.^21,22^ However, a recent study found that PEP can contribute to even more than expected outcomes.^23^ Other studies have found that PEPs are well perceived by students to increase their knowledge about SRH.^17^ In the present study, the SRH PEP had significantly improved knowledge among school adolescents. These findings are consistent with other studies conducted in other countries.^24,25,26,27^ This may be the case because when young people are taught in classrooms with their peers, they may learn more since they feel more at ease discussing ideas with their peers.^28^

Based on our findings, the mean attitude score on contraceptive methods was significantly improved after the intervention. These results are consistent with other studies which were conducted in other settings.^26,27,29^ In contrast, findings from a study which has been conducted in Malaysia revealed that there was a decrease of participants with positive attitudes toward sexuality with an increase of participants with positive attitudes toward SRH.^30^ Even though there is a mixture of findings, SRH PEP may be a promising strategy to improve the SRH attitudes among the schools’ adolescents.

Most significantly, peer education may provide a safe environment for young people to discuss significant life concerns without fear or outside pressure. This approach is considered advantageous since it makes it easier for people of similar ages to share sensitive information. Similar to this, the theory of social awareness proposes that peers frequently imitate the behaviour of those they hold up as role model.^31^ Therefore, our findings support the usefulness of peer education in raising young people’s knowledge and the attitude of SRH-related issues. Thus, the prevention of risky behaviours can benefit greatly from peer education.

Students from Seniors 5 and 6 had significantly higher knowledge scores compared to those in Seniors 3 and 4 in both the pre-intervention and the post-intervention. Lower secondary school has been determined to contribute to poor knowledge regarding SRH in Cameroon.^32^ This may be because young people who stay in school longer may have more exposure to SRH information through their curricula. Following the intervention, adolescents aged from 22 years to 24 years demonstrated a significantly higher overall knowledge score; however, no significant differences were observed between age groups prior to the intervention. These findings are in contrast with a study which found that age was not associated with an increase in knowledge and attitudes after a PEP in Nigeria.^33^ Increased knowledge among upper years and those aged between 22 years and 24 years in this study might be related to the significant exposure of SRH information in biological courses as they are in options with biology as a principal subject. However, contrary to other studies, there was no association between gender and knowledge of SRH in both before and after the interventions.^24,34^

The key strength of this study is that the intervention was designed in collaboration with several stakeholders to assess the local needs and contexts. As a result, we developed a manual which is tailored to adolescents’ needs. The implementation was well monitored with series of qualitative surveys. Constant communication between the project team and the schools helped to address any challenges which could hinder the implementation as soon as possible. However, this study has limitations. Firstly, in post-test we did not consider the third school because of the challenges faced during the implementation, including relocation of mentors to other schools and repatriation of PEs to their country of origin. Students from this school might have different responses. Secondly, the sample size was small to the extent that findings could not be generalised.

## Conclusion

This study showed that SRH PEP is a crucial method for educating adolescents and young adults about sexual and reproductive health. Significant positive changes in knowledge and attitudes were observed following SRH PEP intervention. In order to provide teenagers enrolled in schools with accurate and trustworthy SRH information, a well-designed SRH PEP is a successful approach. Further study is also recommended to investigate the effect of the SRH PEP on reducing risky sexual practices.
